# Expanded CD133^+^ Cells from Human Umbilical Cord Blood Improved Heart Function in Rats after Severe Myocardial Infarction

**DOI:** 10.1155/2018/5412478

**Published:** 2018-04-11

**Authors:** Alejandro Correa, Gabriel Salles Ottoboni, Alexandra Cristina Senegaglia, Luiz Guilherme Achcar Capriglione, Nelson Itiro Miyague, Lidiane Maria Boldrini Leite, Valderez Ravaglio Jamur, Carmen Lúcia Kuniyoshi Rebelatto, Márcia Olandoski, Paulo Roberto Slud Brofman

**Affiliations:** ^1^Carlos Chagas Institute, Oswaldo Cruz Foundation, FIOCRUZ, Curitiba, PR, Brazil; ^2^Core for Cell Technology, Pontifícia Universidade Católica do Paraná, Curitiba, PR, Brazil

## Abstract

Pharmacological approaches are partially effective in limiting infarct size. Cell therapies using a cell population enriched with endothelial progenitor cells (EPCs) CD133^+^ have opened new perspectives for the treatment of ischemic areas after infarction. This preclinical study evaluated the effect of intramyocardial transplantation of purified or expanded human umbilical cord blood-derived CD133^+^ cells on the recovery of rats following acute myocardial infarction (AMI). Histology studies, electrocardiogram, and fluorescence in situ hybridization (FISH) were used to evaluate heart recovery. Purified CD133^+^ cells, enriched in endothelial progenitor cells, when expanded *in vitro* acquired an endothelial-like cell phenotype expressing CD31 and von Willebrand factor (vWF). The group of infarcted rats that received expanded CD133^+^ cells had a more significant recovery of contraction performance and less heart remodeling than the group that received purified CD133^+^ cells. Either purified or expanded CD133^+^ cells were able to induce neovascularization in the infarcted myocardium in an equivalent manner. Few human cells were detected in the infarcted myocardium of the rats 28 days after transplantation suggesting that the effects observed might be related primarily to paracrine activity. Although both cell populations ameliorated the infarcted heart and are suitable for regeneration of the vascular system, expanded CD133^+^ cells are more beneficial and promising candidates for vascular regeneration.

## 1. Introduction

Despite advances in the diagnosis and treatment of acute myocardial infarction (AMI), this cardiovascular disease continues to have a major impact on public health [1]. Although mortality has decreased by approximately 30% in recent decades, AMI incidence is still a fatal event in approximately one-third of patients. The vast majority of the cases result from coronary atherosclerosis and superimposed thrombosis. The fissure and the consequent rupture of atherosclerotic plaque is currently considered the common pathophysiological basis of the onset of symptoms [2].

Following occlusion of a coronary artery, the surrounding myocardial muscle area enters an ischemic cascade and loses its contractile function. Compensatory mechanisms are activated to restore ventricular function and cardiac output. However, myocardial fibrosis and changes in the thickness of the ventricular wall lead to cardiac remodeling and the loss of ventricular cavity dilation function [3].

Current pharmacological approaches are partially effective in limiting infarct size [4]. Restoring myocardial perfusion represents one way to normalize blood circulation and oxygen demand. Intravenous thrombolysis with thrombolytic agents also plays an important role in the treatment of AMI. This therapy is effective in rechanneling coronary occlusion by a thrombus [5]. However, percutaneous coronary angioplasty is currently the gold standard treatment for acute myocardial infarction [6], whereas only selected cases are candidates for surgical treatment [7].

Recently, a new therapy is being studied at the clinical level, aiming to treat patients with myocardial infarction and to make up for the time that is lost prior to revascularization. Cell therapies using CD133^+^ cell population enriched with endothelial progenitor cells (EPCs) have opened new perspectives for the treatment of ischemic areas after infarction [8–13].

In a previous study, we characterized and evaluated the angiogenic potential of CD133^+^ cells and speculated that expanded CD133^+^ cells might have clinical advantages over purified CD133^+^ cells for treating AMI [14]. In this work, we carried out an in-depth study and show that in fact infarcted rats treated with expanded CD133^+^ cells have less mortality, significantly improved ejection fraction, significantly less ventricular remodeling, and more mature vascularization than those treated with purified CD133^+^ cells. The low number of human CD133^+^ cells found in the heart after 28 days of treatment suggests that the improvements observed were primarily due to the paracrine effectors secreted by these cells.

## 2. Materials and Methods

This animal study and the procedures detailed herein were reviewed and approved by the Local Ethics Committee on Animal Research, identification number 180. Signed informed consent was obtained from each mother prior to human umbilical cord blood (HUCB) collection.

### 2.1. Purification and Expansion of Endothelial Progenitor Cells (EPCs)

The experiments were conducted with samples of human umbilical cord blood obtained at Hospital Victor Ferreira Amaral from mothers who agreed to participate in the study. Under sterile conditions, HUCB was collected from fresh placentas with the umbilical cord still attached. The puncture was performed with 60 and 20 ml syringes using the anticoagulant acid citrate dextrose (ACD) (JP Indústria Farmacêutica S.A., Ribeirão Preto, Brazil) after the suspension of the placenta.

The isolation of mononuclear cells (MNCs) was performed according to the method of Boyum [15] modified using a Histopaque™ 1.077 density gradient (Sigma-Aldrich, São Paulo, Brazil). EPCs (CD133^+^) were selected using CD133-coupled magnetic microbeads (Miltenyi Biotec, Bergisch Gladbach, Germany) according to manufacturer's instructions. The purity of the MACS-separated subpopulations was confirmed by flow cytometry with monoclonal antibodies (CD34, CD45, and CD133). After isolation, CD133^+^ cells were expanded as described elsewhere by Senegaglia et al. [14]. Briefly, isolated CD133^+^ cells were plated in 25 cm^2^ flasks in Iscove's modified Dulbecco's media (IMDM) (Invitrogen, Grand Island, NY, USA) supplemented with 50 ng/ml vascular endothelial growth factor (VEGF) (Sigma-Aldrich, São Paulo, Brazil), 1 ng/ml basic fibroblast growth factor (b-FGF) (Sigma-Aldrich, São Paulo, Brazil), 2 ng/ml insulin-like growth factor (IGF)-I (Gibco Invitrogen, Carlsbad, USA), 10% fetal bovine serum (FBS) (Gibco Invitrogen, Carlsbad, USA), and 1% penicillin-streptomycin (Gibco Invitrogen, Carlsbad, USA). All cultures were maintained at 37°C with 5% CO_2_ in a humidified atmosphere. After MACS sorting, the number of CD133^+^ cells obtained was low (in average 6.6 × 10^5^ cells) and approximately 20 days of culturing were necessary to achieve confluence. After that, cells were passed every 3-4 days. To obtain enough number of cells for the *in vivo* experiments, they were used at passage 4 or 5. At the same time, this expansion is sufficient for the cells to acquire an endothelial-like phenotype [14], yet maintaining a young and highly proliferative cell population with no changes in their properties or in the genetic stability of the cells. For intramyocardial infusion, cells were prepared in a 1 ml syringe, and 2 × 10^5^ cells were diluted in 0.3 ml isotonic saline (sodium chloride 0.9%) (JP Indústria Farmacêutica S.A., Ribeirão Preto, Brazil). In the control group, the syringes contained only 0.3 ml isotonic saline (0.9% sodium chloride).

### 2.2. Characterization of the Cells

Immunophenotypic analysis was performed by staining 2 × 10^5^ MNCs, purified and expanded CD133^+^ cells per tube. MNCs and purified cells were analyzed after isolation, whereas expanded cells were analyzed at passage 3 or 4. Anti-mouse IgG1 antibodies conjugated with phycoerythrin (PE), fluorescein isothiocyanate (FITC), allophycocyanin (APC), and peridinin chlorophyll protein (PerCP) (all from BD Pharmingen™ San Jose, CA, USA) were used as isotype controls. The cells were incubated with the following monoclonal antibodies to determine their typical cell surface epitope profiles: anti-CD14, anti-CD31, anti-CD34, and anti-CD45 (all from BD Pharmingen™ San Jose, CA, USA), anti-CD105 (eBioscience Inc., San Diego, CA, USA), and anti-CD133 (Miltenyi Biotec, Bergisch Gladbach, Germany). For intracellular detection of vWF, cells were permeabilized using FIX & PERM cell permeabilization reagents (Caltag, Carlsbad, CA, USA) and further incubated with an isotype-specific FITC-conjugated goat anti-rabbit antibody (Sigma-Aldrich, São Paulo, Brazil). Their viability was assessed by 7-AAD (BD Pharmingen) staining. The data for cell staining were acquired using a FACSCalibur flow cytometer (Becton Dickinson, San Jose, CA, USA) and analyzed with FlowJo software (Tree Star, Ashland, OR, USA).

### 2.3. Experimental Model of the AMI in Rats

The study included 38 male 100-day-old albino Wistar rats *(Rattus norvegicus)* (mean weight of 342 grams). Male animals were used to avoid the hormone cycle of the female rats. The hormone cycle would add one more variable to our study. The rats were housed in open-top polypropylene cages (41 cm × 34 cm × 16 cm (height)) in groups of three or four rats/cage in a temperature- (18–21°C) and humidity-controlled (55–65% relative humidity) environment under a 12-hour light-dark cycle and had ad libitum access to a standard rodent chow (NUVITAL®, Colombo, Paraná, Brazil) and water. The bedding (pine wood shavings, Inbrasfama, São José dos Pinhais, Paraná, Brazil) in each cage was changed daily.

This animal model was performed according to the methodology described by Capriglione et al. [16]. For anesthetization, the rats were first premedicated by intraperitoneal (IP) injections of 1.25 mg/kg diazepam (Valium®, 5 mg/ml, Teuto, Anápolis, Goiás, Brazil) and 12.5 mg/kg ketamine (Vetanarcol®, 50 mg/ml, Laboratórios König S.A., Avellaneda, Argentina) and an intramuscular (IM) injection of 5 mg/kg meperidine (Dolosal®, 50 mg/ml, Cristália, São Paulo, Brazil) and 40 mcg/kg atropine (SANTROPINA®, Santisa, Brazil, 0.25 mg/ml). Five minutes after the injections, anesthesia was induced using halothane (Tanohalo®, Cristália, São Paulo, Brazil). Each rat was then endotracheally intubated, and anesthetization was maintained by halothane vaporized in 100% oxygen (~150 ml/minute) in a semiclosed breathing circuit. Halothane delivery to the anaesthetized rats was not continuous: it was stopped at the time of left coronary artery occlusion or when the rat was at the desired depth of anesthesia. Each rat was mechanically ventilated using a ventilator (Harvard model 683 small animal ventilator, Harvard Apparatus, MA, USA), which was set to 70–80 breaths/minute and a minute volume of 175–200 ml/min.

For surgery, the animal was placed in dorsal decubitus position, and a lateral thoracotomy was performed in the left fourth intercostal space, separating the latissimus dorsi and pectoral muscles. The intercostal space was kept open using a 7 cm AlM self-retaining retractor to visualize the beating heart. The pericardium was then opened using a sterile flexible cotton-tipped rod. The left anterior descending coronary artery (LADCA) was first identified and then occluded 2 mm from its origin between the left atrial edge and the pulmonary artery sulcus using 7-0 polypropylene thread (Prolene®, Ethicon Inc., Somerville, USA). The infarcted area was immediately verified by the color difference and loss of contractile function. The thorax was then closed in two layers with simple interrupted 4-0 monofilament nylon sutures. Each rat was returned to its home cage after it fully recovered from the anesthesia and surgery and was kept under the laboratory standard conditions (see previous). After surgery, the rats received flunixin meglumine anti-inflammatory (Banamin®, São Paulo, Brazil) 2.5 mg/kg/SC twice daily for 2 days and enrofloxacin antibiotic (Baytril 5%®, São Paulo, Brazil) 10 mg/kg/IM once daily for 2 days. The surgical wounds were cleansed with isotonic saline (sodium chloride 0.9%) once daily until complete healing, and the suture was removed after 7–10 days.

### 2.4. Echocardiographic Evaluation

Transthoracic echocardiography (TTE) was performed seven days after AMI and 28 days after cell transplantation by an experienced professional who did not have knowledge of the experimental groups.

For TTE, the rats were sedated by an IM injection of 50 mg/kg ketamine and 5 mg/kg xylazine (KENSOL® König, Brazil, 20 mg/ml). When sedated, they were placed in the dorsal decubitus position with the body slightly inclined to the left. Two-dimensional TTE was performed using a multifrequency linear array ultrasound transducer (15L6, bandwidth 15 MHz, Philips Ultrasound, USA) whose output was recorded on a Hewlett Packard Sonos 5500 Ultrasound System. Ejection fraction (LVEF), end-systolic volume (ESV), end-diastolic volume (EDV), end-systolic area (ESA), and end-diastolic area (EDA) of the left ventricle were determined from the images using Simpson's method [17]. The heart rate (HR) of these rats was simultaneously measured by an electrocardiograph that was incorporated into the ultrasound system. All echocardiographic measurements were performed using the same equipment and were repeated three times by the same examiner. The results are represented as the mean of three independent measurements.

Rats with ejection fractions less than 40%, featuring ventricular dysfunction, were included in further experiments.

### 2.5. Cell Transplantation

On the ninth day after AMI, the rats were anesthetized with the same protocol and details previously described (see Experimental Model of the AMI in Rats). After antisepsis of the anterior chest region, the rats were placed in a dorsal decubitus position and a median thoracotomy was performed. The infracted region of the left ventricle was visualized by the staining difference and scarring. The cell transplants (purified or expanded) or isotonic saline injections (IS) were performed by a single injection to the central area of the AMI. The cell transplant was performed using a 1 ml insulin syringe (13 mm × 0.38 mm needle) containing 2 × 10^5^ stem cells diluted in 0.3 ml IS. In the control group, the same syringe was used with the needle containing only 0.3 ml IS. The chest walls were closed via simple suture using catgut 4.0 in the intercostal muscle and in the skin with nylon monofilament 4.0. After recovery from anesthesia, the rats were treated with the same anti-inflammatory and antibiotics utilized previously (see Experimental Model of the AMI in Rats) and were then returned to their home cages where they were kept for 28 days under the conditions described in Experimental Model of the AMI in Rats. The rats were also followed up daily for clinical signs of illness and behavioral problems, such as aggression or stereotypic behaviors. After 28 days, the second TTE was performed followed by euthanasia. The rats were humanely killed without the presence of other rats by an overdose of halothane after being placed in the glass induction chamber that was used to induce anesthesia. After confirmation of death, each rat was necropsied.

### 2.6. Histopathology of the Hearts

After euthanasia, each rat was necropsied and the heart of each one was removed for histopathological analysis. The hearts were fixed in a 10% neutral buffered formalin solution (Biotec, Pinhais, Brazil) for 24 hours. Briefly, the formalin-maintained samples were washed in tap water, dehydrated using an ascending alcohol series, and then embedded in paraffin blocks. Sections (5 *μ*m thick) were cut, mounted on glass slides, hydrated using distilled water, and then stained. Hematoxylin and eosin staining of cardiac tissue was performed to locate the infarct and to evaluate the formation of new capillaries in the infarcted area, and then the neovascularization findings of the three groups were compared. To analyze the estimated quantification of capillaries, slides were examined under an Olympus CX41 microscope with a camera (model DP25) (Olympus, São Paulo, Brazil) attached to the microscope. The images were captured using the analySIS getIT program (software installed on Windows). We used a field of 100 *μ*m at a magnification of 20x. The hearts were submitted to three cross sections, and the most significant area of AMI was selected. At an objective of 10x, three regions of infarct, including one central (larger infarct size) and two peripherals (the transition region between the infarct and the healthy area) regions, were selected. In each of the three selected regions, six random fields at the 20x objective were chosen, and then the capillaries were counted. A capillary was defined as a tubular histological structure with at least one endothelial cell with a nucleus and cytoplasm in a circular shape and containing at least one red blood cell in the interior and no middle layer muscle.

We counted the capillaries in 18 fields of each slide. Twenty slides were analyzed per group. Three investigators performed the counts, and the results were expressed as the mean of the capillaries per field in the peripheral, central, and total areas for all three groups. The same histological slides used to count the capillaries were used to determine the relative amount of fibrosis via a semiquantitative analysis of the infarcted region of the heart using what we have named as the fibrosis value (F.V.). A maximum value of four (F.V. = 4) was assigned to the worse cases of fibrosis observed in the analyses, and a value of 0 (F.V. = 0) represented the absence of fibrosis (healthy muscle heart). More specifically, the scale was defined as follows: 0—preserved heart tissue without histological changes; 1—thinning of the ventricular wall, fibrosis deposition in localized regions; 3—thinning of the ventricular wall, fibrotic deposition in diffused regions but with the presence of some preserved cardiac tissue; and 4—very decreased thickness of the ventricular wall, intense deposition of collagen throughout the infarcted region. All the analyses were carried out such that the experimenters were blinded to the identity of the samples.

### 2.7. Analysis of Transplanted Cells In Situ

Paraffin-embedded tissue sections for FISH were prepared according to Henegariu [18]. Briefly, the heart tissue sections were maintained for one hour at 60°C, placed in xylene (Merck, Darmstadt, Germany) for 10–15 minutes, placed in a xylene/ethanol (Merck, Darmstadt, Germany) solution (1 : 1) for ten minutes, and finally, placed in 100% ethanol for ten minutes. Sections were then treated with proteinase K solution (20 ml phosphate-buffered saline (PBS) (Invitrogen, Carlsbad, USA) + 100 *μ*l 10% sodium dodecyl sulfate (SDS) (Sigma-Aldrich, Steinheim, Germany) + 200 *μ*l proteinase K (Invitrogen, Carlsbad, USA) 20 mg/ml) for 8 to 25 minutes at 45°C, then washed with PBS for 3 minutes, and finally dehydrated with ethanol solutions of 70% and 100% for five minutes each. The prepared slides were dehydrated again with an ethanol sequence of 70%, 85%, and 100% for two minutes each. Tissue sections were placed in each slide with a probe for all human centromeres (AHC) shown in red (Kreatech Diagnostics, Amsterdam, Netherlands) and a probe for rat chromosome Y shown in green (ID Labs Inc., London, Canada). Codenaturation, hybridization, and washing were performed according to manufacturer's instructions. To display the signal, DAPI (Kreatech Diagnostics, Amsterdam, Netherlands) was used as a counterstain, which has an affinity for genetic material. The images were captured using a LAS system (Leica Microsystems, Mannheim, Germany) with green, red, blue, and overlapping filters.

### 2.8. Statistical Analysis

A one-way analysis of variance (ANOVA) was used to compare the groups with respect to the quantitative variables of the echocardiographic parameters that were assessed pretransplantation. The nonparametric Kruskal-Wallis test was used to compare the percentage of collagen and the number of capillaries among groups. Student's *t*-test for paired samples was used to compare echocardiographic parameters pre- and posttransplantation. The results obtained are expressed as the mean ± SD or as the median, minimum, and maximum values. *p* values < 0.05 were considered statistically significant. All statistical analyses were performed using SPSS v. 20.0 software.

## 3. Results

### 3.1. Expanded CD133^+^ Cells Acquire an Endothelial-Like Phenotype

**Figure 1:**
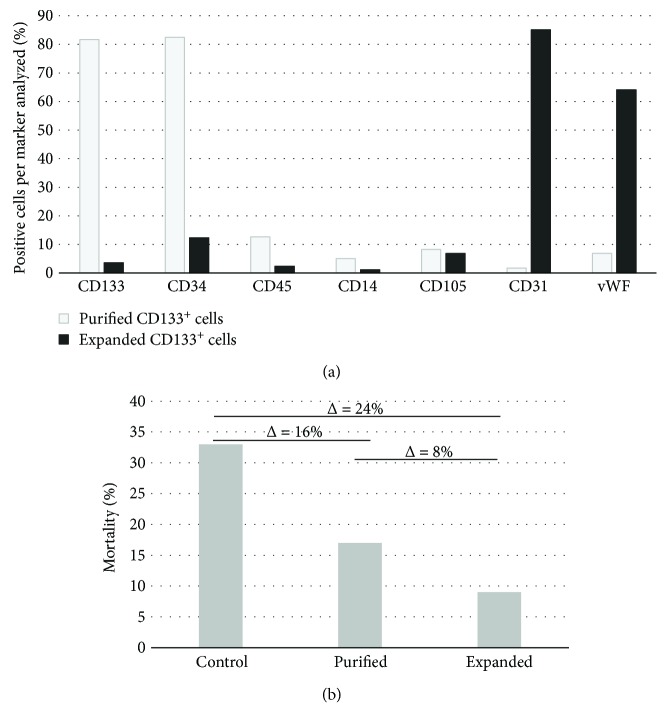
Expanded CD133^+^ cells acquired an endothelial-like immunophenotype and greatly diminished the mortality of rats with AMI. (a) Percentage of positive cells with progenitor and endothelial cell markers. Representative immunoprofile of a sample of CD133^+^ cells isolated from human umbilical cord blood before (purified CD133^+^ cells) and after expansion (expanded CD133^+^ cells). (b) Percentage of mortality of the three groups: control (injected with isotonic saline solution, sodium chloride 0.9%), purified (transplanted with purified CD133^+^ cells), and expanded (transplanted with expanded CD133^+^ cells). The differences between groups are expressed as the delta value (Δ) above the bars.

The average volume of blood collected from eleven human umbilical cord samples was 93 ± 40 ml. The average number of mononuclear cells obtained was 70 ± 78 × 10^6^ and the average number of CD133^+^ cells was 0.66 ± 0.62 × 10^6^, about 0.95% of the total mononuclear cell population. After culturing the CD133^+^ cells for 3 to 4 passages in IMDM containing b-FGF/IGF-1/VEGF, the average number of expanded cells was 1.99 ± 0.89 × 10^6^ (Table S1); thus, a mean increase of three times the number of cells was observed comparing expanded to purified cells. The correlation coefficients between the volume of blood collected versus the number of mononuclear cells, the number of mononuclear cells versus the number of CD133^+^ cells, and the number of CD133^+^ cells versus the number of expanded CD133^+^ cells were 0.69, 0.84, and 0.32, respectively. As it has been previously shown by our group [14, 19] that the phenotype of the enriched CD133^+^ cell population, as determined by surface markers, changes considerably after expansion, acquiring an endothelial-like phenotype. After isolation, the average percentages of cells were as follows: 81% CD133, 82% CD34, 12% CD45, 5% CD14, 8% CD105, 1% CD31, and 6% vWF. The phenotypes of the expanded CD133^+^ cell population were on average as follows: 3% CD133, 12% CD34, 2% CD45, 1% CD14, 6% CD105, 85% CD31, and 64% vWF (Figure 1(a)).

### 3.2. Expanded CD133^+^ Cells Improve Cardiac Function of the Infarcted Heart and Prevent Major Heart Remodeling More Effectively Than Purified CD133^+^ Cells Alone

**Figure 2:**
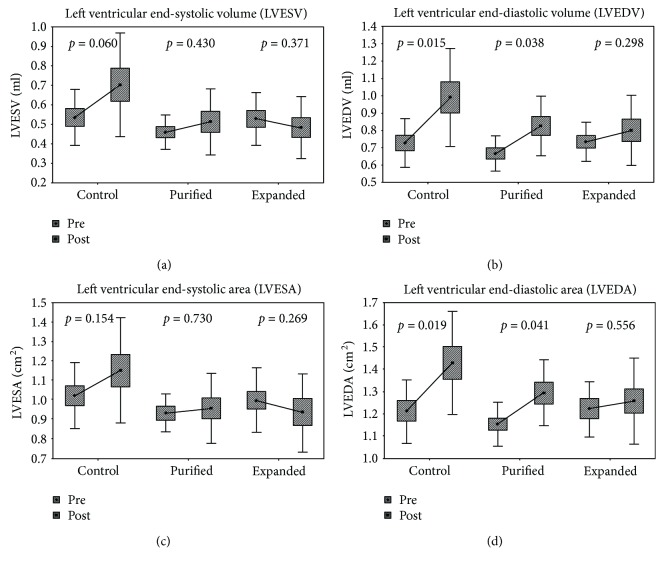
Echocardiographic evaluations revealed better-preserved hearts in the group of animals transplanted with expanded CD133^+^ cells. Comparison of left ventricular end-systolic volume (a), left ventricular end-diastolic volume (b), left ventricular end-systolic area (c), and left ventricular end-diastolic (d) within each group pretreatment and posttreatment. The results are presented as the mean (point in the box) with the standard error (stripped boxes) and standard deviation (lines). Control = control group, injected with isotonic saline solution (sodium chloride 0.9%). Purified = transplanted with purified CD133^+^ cells. Expanded = transplanted with expanded CD133^+^ cells. The *p* values of each comparison are shown on top of the boxes.

The rats with AMI included in this study were divided into three groups as follows: a control group (C) that received saline solution (*n* = 15), a group transplanted with purified CD133^+^ cells (P, *n* = 12), and a group transplanted with expanded CD133^+^ cells (E, *n* = 11). In the control group, 33% (5 of 15) of the mortality was observed after surgery during the 28 days of follow-up. In the group that received purified CD133^+^ cells, 17% (2 of 12) of the rats died during treatment, while in the group that received expanded CD133^+^ cells, mortality dropped to only 9% (1 of 11) compared with the control group (Figure 1(b)). Nevertheless, no significant differences in mortality rate were observed among groups. To evaluate heart functionality and remodeling, several cardiovascular parameters were determined to establish statistical similarities or differences among groups. The values related to heart rate (HR) decreased after 28 days posttransplantation in all three groups, albeit no statistic differences were observed within or among groups (Table S2). The end-systolic volumes of the left ventricle (LVESV) pre- and posttransplantation were analyzed in the three groups. In the control group, rats exhibited a trend of systolic dilatation (*p* = 0.06). The average volumes pretransplantation and 28 days posttransplantation were 0.53 ml and 0.70 ml, respectively. On the other hand, there was a clear preservation of LVESV in the others treated groups. The LVESV of the group that received the purified CD133^+^ cells fluctuated from 0.46 ml to 0.51 ml (*p* = 0.43), and that of the group transplanted with the expanded cells ranged from 0.53 ml to 0.48 ml (*p* = 0.37). No significant differences were observed in the LVESV of the posttransplanted rats between groups P and E (*p* = 0.750), while the differences between P and C (*p* = 0.047) and between E and C (*p* = 0.023) (Figure 2(a) and Table S3) were significant. For end-diastolic volume of the left ventricle (LVEDV), the group C showed a significant increase in LVEDV comparing pre- and posttransplanted rats ranging from 0.73 ml to 0.99 ml with a *p* value of 0.015. Group P also showed a significant increase from 0.67 ml to 0.83 ml (*p* = 0.038), suggesting dilation of the left ventricle in both the C and P groups. Conversely, group E did not show a significant increase in LVEDV, ranging from 0.73 to 0.80 ml (*p* = 0.298), and thus the left ventricle was preserved (Figure 2(b) and Table S4).

The average values of the end-systolic area of the left ventricle (LVESA) for the pre- and posttransplanted rats were compared, and no significant differences were observed within each group (Figure 2(c) and Table S5). In addition, at the end of the 28 days of follow-up, no significant difference was observed in the LVESA values among groups. In contrast, the data of the left ventricular end-diastolic area (LVEDA) for the pre- and posttransplanted rats were significantly different between groups C (pretreatment = 1.21 cm^2^ and posttreatment = 1.43 cm^2^; *p* = 0.019) and P (pretreatment = 1.16 cm^2^ and posttreatment = 1.30 cm^2^; *p* = 0.041). In group E, the values ranged from 1.22 cm^2^ to 1.26 cm^2^ with *p* = 0.556. Therefore, there was a significant increase in the end-diastolic area of groups C and P, while that of group E was not significantly different (Figure 2(d) and Table S6).

**Figure 3:**
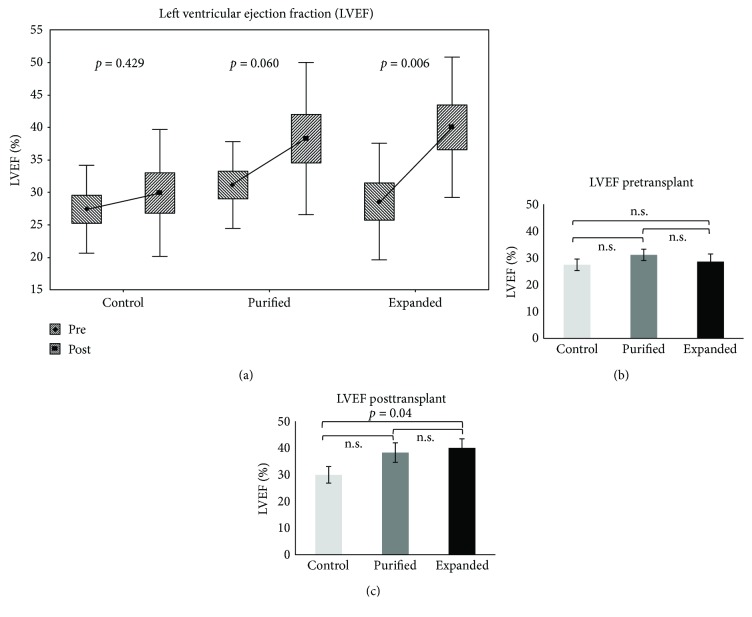
The ejection fraction of the animals with AMI significantly increased after transplantation with expanded CD133^+^ cells. Comparison of the left ventricle ejection fraction within each group (a) and among the groups before (b) and after transplantation (c). Control = control group, injected with isotonic saline solution (sodium chloride 0.9%). Purified = transplanted with purified CD133^+^ cells. Expanded = transplanted with expanded CD133^+^ cells. The *p* value is shown for each comparison.

One of the most revealing parameters of cardiac function is the LVEF. The mean value of the LVEF of healthy Wistar rats has been well established and is approximately 80% [20–22]. In our study, we included rats with EF < 40% after infarct, representing sever damage in the cardiac function due to considerable areas of noncontractile fibrotic tissue. The LVEF percentages of the control group pre- and posttransplant showed a slight but not significant increase from 27.4% to 29.9% (Δ = 2.5%), *p* = 0.429. In the group of purified CD133^+^ cells, there was a trend toward an improvement in the ejection fraction from 31.1% to 38.3% (Δ = 7.2%, *p* = 0.06), although the difference was not significant. Remarkably, the group transplanted with expanded CD133^+^ cells exhibited a significantly increased LVEF from 28.6% to 40.0% (Δ = 11.4%, *p* = 0.006) (Figure 3(a) and Table S7). It is important to note that no differences were observed among the groups before transplant (*p* = 0.538, Figure 3(b)); however, the posttransplantation group that received expanded CD133^+^ cells was significantly different compared with the control group (*p* = 0.04, Figure 3(c) and Table S7).

### 3.3. Transplant of Either Expanded or Purified CD133^+^ Cells Decreases Fibrosis and Significantly Increases Vascularization in Myocardial Tissue after Infarct

**Figure 4:**
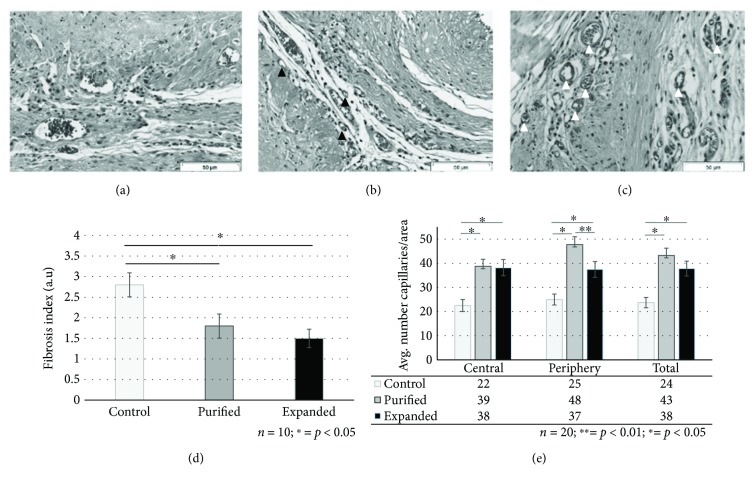
Either purified CD133^+^ cells or expanded CD133^+^ cells decreased fibrosis and increased angiogenesis of rat hearts that underwent AMI. Representative photomicrographs of myocardial tissue sections in the infarct region of the three groups: control (a), transplanted with purified CD133^+^ cells (b), and expanded CD133^+^ cells (c). Control = control group, injected with isotonic saline solution (sodium chloride 0.9%). Purified = transplanted with purified CD133^+^ cells. Expanded = transplanted with expanded CD133^+^ cells. Semiquantitative analyses of cross-sectional areas were carried out to determine the level of fibrosis in the infarcted region of the heart (d) and the level of vascularization in the central/periphery regions of the heart (e) (for further details, see Materials and Methods). The control group presented less capillaries than the groups treated with CD133^+^ cells. The presence of many well-developed vessels was observed in animals transplanted with expanded CD133^+^ cells (white arrowheads in (c)). Structures resembling recently formed capillaries with one or without red blood cells were identified in rats transplanted with purified CD133^+^ cells (black arrowheads in (b)). The bars in graphs (d) and (e) represent the mean ± the standard error. Scale bars: 50 *μ*m.

To determine the level of fibrosis in the cardiac tissue, a semiquantitative microscopic analysis was carried out (for further details, see Materials and Methods). In the control group that did not receive cell therapy, the cardiac tissue maintained a typical AMI pattern with large areas of fibrosis between the myocardial tissue islands (F.V. = 2.8; SD = 1.03). Additionally, it was possible to observe less capillaries in the control group than in the transplanted groups and no evidence of new vascular-like structures (circular or in networks) that suggested capillary formation (Figures 4(a) and 4(d)). In group P, larger areas of myocardial tissue in islands were identified mixed with fibrosis regions compared with group C (F.V. = 1.8; SD = 0.89). Numerous capillaries arranged in the form of vascular beds in the transplanted area were observed. Concomitantly, structures resembling capillary formation in various stages of organization were evident both with and without red blood cells inside (Figures 4(b) and 4(d)). Remarkably, the hearts of group E showed fewer regions of fibrosis (F.V. = 1.53; SD = 0.53) than those of group P, although the difference was not statistically significant. In comparison with the control group, the fibrosis in E group was markedly reduced. Additionally, the presence of many well-developed vessels and few structures resembling recently formed capillaries without red blood cells was observed. The capillaries showed a layout pattern that resembled more mature networks than those observed in group P (Figures 4(c) and 4(d)).

To assess the degree of revascularization of the ischemic tissue, a quantitative analysis was carried out by identifying and counting the capillaries of the infarcted and peri-infarcted areas in the three groups. The mean number of capillaries in the central region of the infarct area was 22.5 in group C, 38.7 in group P, and 38.2 in group E. In the peripheral area, the mean number of capillaries was 25.0 for group C, 47.8 for group P, and 37.5 for group E. When assessing the total region including the infarct and periphery, the capillary average per area was 23.7 for group C, 43.2 for group P, and 37.8 for group E, indicating a beneficial angiogenic effects of cell transplantation either with purified or expanded CD133^+^ cells (Figures 4(a)–4(c) and 4(e)). To gain information on capillary maturity, a morphology analysis comparing purified and expanded CD133^+^ groups was performed, which suggested different patterns among groups. Specifically, the capillaries observed in group E had a more developed morphology in comparison with the capillaries in group P. Capillaries in the group that received purified CD133^+^ cells had a smaller caliber and were often formed by a single endothelial cell containing a single erythrocyte cell. The capillaries in group E were larger in size, were formed by more endothelial cells (two to four cells), and contained many erythrocytes (>5) (Figure 4(e)).

### 3.4. Few Transplanted CD133^+^ Cells Remained in the Infarcted Cardiac Tissue after 28 Days

To determine if human purified or expanded CD133^+^ cells transplanted into the central area of AMI via a single intramyocardial injection were still present in the region 28 days after transplantation, FISH analyses were carried out using a human pancentromere-specific probe. Transplanted cells were identified in both cell groups, P and E. In the control group, eight rats were tested and no FISH-positive cells were observed (Figure S1A). In five of the nine tested rats of the group P (Figure S1B) and in five of the eight rats of the group E (Figure S1C), human cells were observed. In all cases, positive human cells were scarce, and as a result of sample processing for FISH analysis, it was not possible to discern if the human cells were integrated into the blood vessels.

## 4. Discussion

Although somehow controversial [23–25], purified CD133^+^ cells are enriched in endothelial progenitor cells in comparison with the original mononuclear cell population, and in addition, expanded CD133^+^ cells have increased the expression of markers CD31^+^ and vWF^+^, typical of endothelial-like cells [14, 19]. Most reports that have used the CD133^+^ cell population [11, 26–28] or expanded endothelial progenitors [12, 19, 29] for treating heart infarcts in animal models have shown some type of improvement regarding heart function and vascularization with variations due to the differences among the studies. Cell source, cell purification, number of transplanted cells, route of transplantation, and time of treatment after infarction may influence the effectiveness of the treatment. Even total mononuclear cell population containing only 1.5% of endothelial progenitors [9, 30] was shown to exert beneficial effects on heart function after infarction. No previous study analyzed several cardiac parameters in parallel, fibrosis level, neovascularization, and engraftment comparing endothelial progenitor (CD133^high^ CD31^low^) cells and endothelial-like (CD133^low^ CD31^high^) cells derived from the same source. The echocardiographic results showed a more effective and significant recovery of the left ventricular function in the group treated with adult endothelial-like cells than in the group treated with progenitor cells. Most infarcted rats in the group regained ventricular function.

Albeit all the rats had a LVEF < 40% before transplantation, the infarct model used in this work results in a wide variation of ejection fractions. The LVEFs ranged from approximately 25% to 40% in all groups. Remarkably, even given this variation in the experimental groups before transplantation, the rats that received expanded CD133^+^ cells showed not only a robust improvement in LVEF but also a preservation of end-diastolic dimensions, preventing dilation and remodeling. Regarding end-systolic volume and area, prevention of remodeling was observed in both treated groups. Thus, considering the entire set of results, the hypothesis raised in our previous publication [14] stating that expanded CD133^+^ cells would be better candidates than purified cells for cardiac infarct treatment was confirmed in the present study.

In accordance with previous publications [9, 12, 14, 19, 28, 31, 32], our histological analysis confirmed the induction of neovascularization by both groups of cells in comparison with the control group. Nevertheless, expanded CD133^+^ cells apparently formed well-developed capillaries in comparison to purified CD133^+^ cells, for which signs of immature vascularization were observed. Similar results were obtained by Schuh et al. [31] using cultivated progenitors expressing markers of mature endothelial cells. Neovascularization was also confirmed in Hu's study [9, 12]. The histological analysis and capillary density results revealed a higher level of capillary formation in the group of cultivated EPCs derived from HUCB and transplanted into rats with AMI. In our study, the expanded CD133^+^ cells were maintained in culture for a longer period of time; however, the cell dose was five times smaller. This may suggest that cultivation likely results in the most efficient cell type regarding heart repair.

Asahara et al. [33, 34] showed in a rat model of acute ischemic cardiomyopathy that endothelial progenitor cells participate in the neovascularization processes by migrating and incorporating into ischemic sites, leading to tissue remedial action. However, in our study, CD133^+^ cells derived from HUCB were identified in the infarcted myocardium of rats at extreme low numbers. Thus, it is reasonable to speculate that the human cells transplanted into the rats act mainly in a paracrine manner inducing angiogenesis. Moreover, the functional and histological results seem to indicate that paracrine signaling from expanded CD133^+^ cells is more efficient than purified CD133^+^ cells. It has been shown that CD133^+^ cell expressed secreted angiogenic factors such as vWF [35]. More recently, we have shown that extracellular vesicles from expanded CD133^+^ cell contain proteins [36] and micro RNAs (unpublished data) involved with a variety of angiogenesis-related functionalities. The expansion of CD133^+^ cells results in a more mature endothelial phenotype [14, 32] and this probably alters the profile of secreted factors and the content of extracellular vesicles favoring a more rapid and efficient vascularization of the damage tissue. In accordance with this, expanded but not purified CD133^+^ cells express VEGF mRNA at levels that are detectable by RT-PCR [14]. In addition to preclinical studies, a few recent clinical studies have also been carried out using CD133^+^ cells. These studies have begun to show that the autologous use of CD133^+^ cells appears to be safe and have minor [37] to moderate [38] positive effects for treating injured hearts. Kurbonov et al. [38] recently showed that autologous single intracoronary infusions of purified CD133^+^ cells isolated from bone marrow had a net positive response, reducing the infarct size in patients. Our findings may call for the further expansion of CD133^+^ cells *in vitro* before transplantation.

## 5. Conclusion

Although both cell populations ameliorated the infarcted heart and are suitable for regeneration of the vascular system, expanded CD133^+^ cells are more beneficial and promising candidates for heart/vascular regeneration. In light of the available preclinical and clinical results and the results presented here, we strongly believed that CD133^low^ CD31^high^ (expanded CD133^+^) cells are a promising candidate for future clinical trials.

## Supplementary Materials

Supplementary 1 Table S1: Volume of blood and number of cells collected/purified/expanded from eleven samples of human umbilical cord.

Supplementary 2 Table S2: Heart rate statistics.

Supplementary 3 Table S3: Statistics of the end-systolic volumes of the left ventricle.

Supplementary 4 Table S4: Statistics of the end-diastolic volume of the left ventricle.

Supplementary 5 Table S5: Statistics of the end-systolic area of the left ventricle.

Supplementary 6 Table S6: Statistics of the end-diastolic area of the left ventricle.

Supplementary 7 Table S7: Statistics of the ejection fraction of the left ventricle.

Supplementary 8 Figure S1: Few transplanted human cells were found in the infarcted regions of the heart of the rats after 28 days of treatment. Infarcted heart sections were processed for FISH staining using a human pancentromeric probe (red). Nuclei were stained with DAPI (blue). Representative photomicrograph of the three groups: control/vehicle (A), transplanted with purified CD133^+^ cells (B), and expanded CD133^+^ cells (C). Scale bars: 7.5 *μ*m.
