# Supplementary figures and images for: Expanded CD133+ Cells from Human Umbilical Cord Blood Improved Heart Function in Rats after Severe Myocardial Infarction
Source: Stem Cells Int. 2018 Apr 11;2018:5412478. doi: 10.1155/2018/5412478 (PMC5925035; doi:10.1155/2018/5412478)

## Slide 1
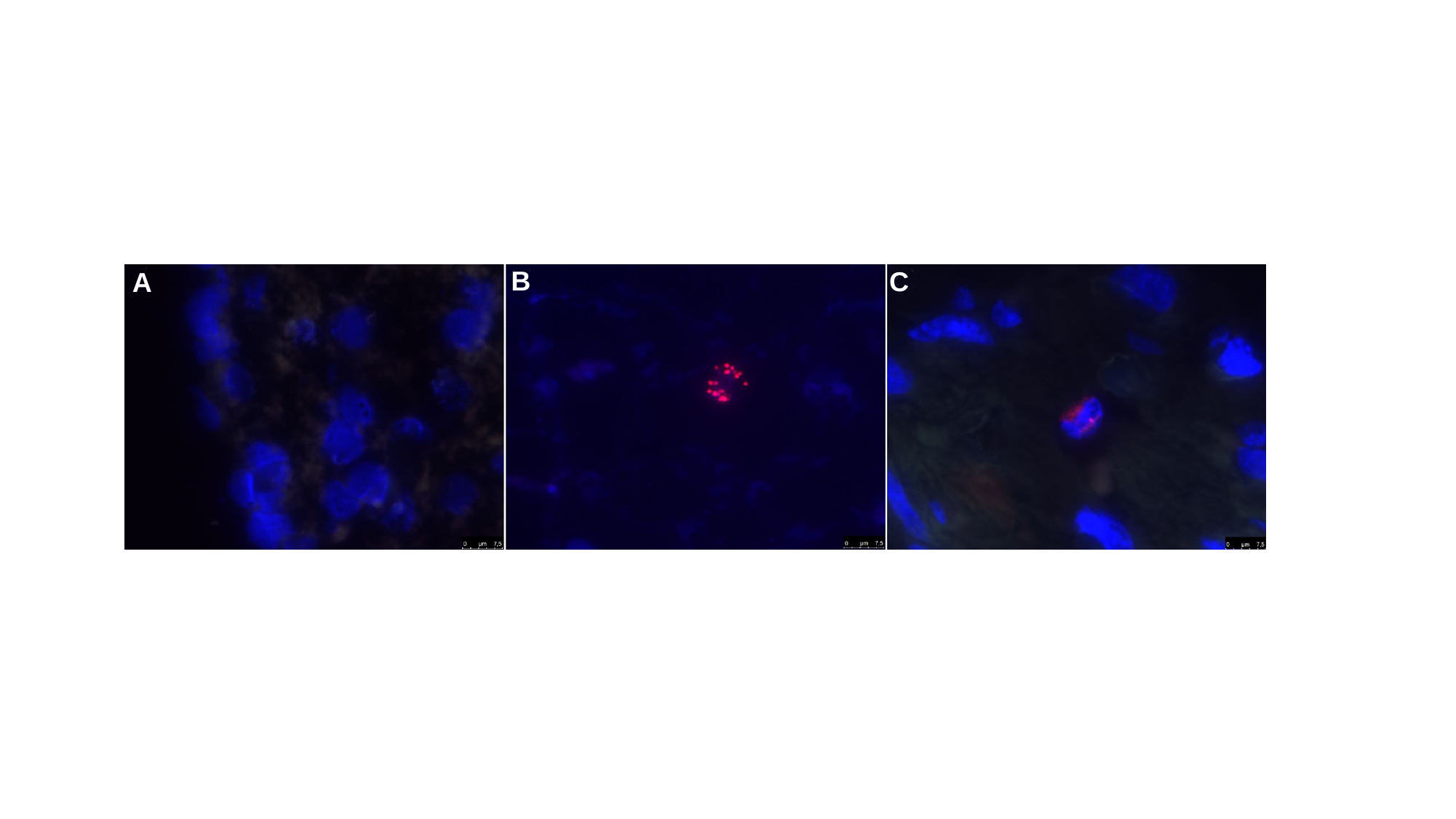

B
C
A

Supplement: Supplementary 8 — Figure S1: Few transplanted human cells were found in the infarcted regions of the heart of the rats after 28 days of treatment. Infarcted heart sections were processed for FISH staining using a human pancentromeric probe (red). Nuclei were stained with DAPI (blue). Representative photomicrograph of the three groups: control/vehicle (A), transplanted with purified CD133+ cells (B), and expanded CD133+ cells (C). Scale bars: 7.5 μm. [file 5412478.f8.pptx]
